# Refractive and Visual Outcomes of Myopic Laser-Assisted In Situ Keratomileusis (LASIK) Centered on the Entrance Pupil Versus 50% of the Pupil-to-Vertex Chord

**DOI:** 10.7759/cureus.117609

**Published:** 2026-10-07

**Authors:** Josh Zhe, Sanjana Molleti, Hanna Pawlowski, Teasha Luu, Konrad C Willey, Kaylyn S Myers, Phillip Hoopes, Majid Moshirfar

**Affiliations:** 1 Department of Ophthalmology, University of Arizona College of Medicine-Phoenix, Phoenix, USA; 2 Hoopes Moshirfar Research Center, Hoopes Vision, Draper, USA; 3 Spencer Fox Eccles School of Medicine, University of Utah, Salt Lake City, USA; 4 College of Medicine, Florida State University, Tallahassee, USA; 5 John A. Moran Eye Center, University of Utah, Salt Lake City, USA; 6 Department of Eye Banking and Corneal Transplantation, Utah Lions Eye Bank, Murray, USA

**Keywords:** 50% offset, ablation, angle kappa, centration, chord mu, laser-assisted in-situ keratomileusis, lasik, pupil center

## Abstract

Background

Laser ablation in myopic laser-assisted in situ keratomileusis (LASIK) is most commonly centered on the entrance pupil, yet the pupil center sits temporal to the visual axis, making the corneal vertex a rational alternative. Comparative studies of the two landmarks have produced conflicting results, and although modern platforms allow the ablation to be placed at any fraction of the distance between them, to the best of our knowledge, no study has compared pupil centration against a 50% offset toward the vertex in myopic eyes. This study compared refractive accuracy, astigmatic outcomes, and the efficacy and safety indices of myopic LASIK centered on the entrance pupil versus 50% of the pupil-to-vertex chord.

Methods

This retrospective cohort study included 389 eyes of 211 patients undergoing primary myopic LASIK by a single surgeon with the WaveLight EX500 excimer laser (Alcon Laboratories Inc., Fort Worth, Texas, USA); 192 eyes centered on the entrance pupil; and 197 eyes treated at 50% of the pupil-to-vertex chord (chord mu). Refractive accuracy, residual astigmatism, Alpins vector indices, and efficacy and safety indices were compared at three months using generalized estimating equations clustered on the patient and adjusted for age, sex, and corneal astigmatism.

Results

Mean chord mu was 210 ± 109 µm and did not differ between groups. No endpoint favored either strategy: 182/192 (94.8%) versus 183/197 (92.9%) of eyes were within ±0.50 D of target (P = 0.56), residual astigmatism was 0.30 D in both groups (P = 0.27), the efficacy index was 1.00 ± 0.15 versus 0.99 ± 0.16 (P = 0.72), the safety index was 1.05 ± 0.13 in both groups (P = 0.95), and no eye lost two or more lines of corrected acuity. Group differences did not vary with chord mu.

Conclusions

In myopic LASIK, no statistically significant differences in refractive or high-contrast visual outcomes were detected between ablation centered on the entrance pupil and ablation centered halfway toward the corneal vertex. The study was not designed as an equivalence trial, and contrast sensitivity, night vision, and aberrometric outcomes were not assessed, so these findings indicate an absence of detected differences rather than established equivalence.

## Introduction

During refractive surgery, corneal laser ablation must be aligned to a reference point on the eye; however, the exact reference point has long been debated. The ablation is typically anchored at one of two locations, either the center of the entrance pupil or the corneal vertex. Modern excimer platforms allow the surgeon to select either or to place the ablation at some fraction of the distance between them, yet nearly two decades of comparative work have not definitively established whether one choice results in better patient outcomes than the other.

Centration on the pupil center became the norm after Uozato and Guyton demonstrated that the best optical result was obtained by centering corneal procedures on the line of sight and pupil center in 1987 [1]. The choice was practical, as the pupil center bounds the rays that enter the eye; it is the reference point for higher-order aberrations, and eye-tracking systems localize the pupil border in real time [1-3]. Subsequent research, however, exposed two weaknesses. The pupil center is not a stable landmark, shifting with illumination, dilation, and body position [4,5]. Moreover, Pande and Hillman showed that the entrance pupil center lies a mean of 0.34 mm (up to 0.75 mm) temporal to the point where the visual axis intersects the cornea, so a pupil-centered ablation can sit substantially off the patient's line of sight [6]. In their measurements, the coaxially sighted corneal light reflex lay closest to that intersection, thus establishing the corneal vertex as the main alternative [6].

Despite these limitations of the pupil center, vertex centration has not replaced it in routine practice, for several reasons. The corneal light reflex is a reproducible but observation-dependent landmark: its apparent position varies with the direction of gaze relative to the light source [7]. Laser platforms compound this issue since eye trackers follow the pupil, not the reflex, so reflex-centered treatment requires the offset to be measured and transferred to the laser rather than tracked directly [2,8]. Optically, the ablation zone is no longer concentric with the pupil when centering on the reflex [3]. While clinically insignificant under normal lighting, the pupil margin risks expanding out of the treatment zone in reflex-centered ablation under mesopic conditions [3]. 

The current literature is heavily mixed on whether a centration strategy affects patient outcomes. Some comparisons have favored ablation shifted toward the vertex, others have suggested possible harm, and pooled analyses have found the two approaches largely equivalent [2,8-10]. Partial offsets were proposed as a means of combining pupil and vertex information rather than choosing between them, on the reasoning that displacing the ablation part of the way toward the vertex brings it closer to the visual axis while preserving more of the zone's concentricity with the pupil than full vertex centration [3]. A 50% offset is the symmetric case of this approach, leaving the treatment center equidistant from the two candidate landmarks, and is selectable on current excimer platforms as a percentage of the measured pupil-to-vertex offset. It has been applied clinically in hyperopes [8], but it has not been compared against pupil centration in myopic eyes. The current study therefore aims to determine whether this intermediate position confers any advantage over pupil centration, comparing refractive accuracy, residual and vectorial astigmatism, and the efficacy and safety indices between pupil-centered laser-assisted in situ keratomileusis (LASIK) and a 50% symmetric offset towards the corneal vertex in myopes at a follow-up of three months.

## Materials and methods

This retrospective study analyzed patients who underwent LASIK in one or both eyes at Hoopes Moshirfar Research Center, Hoopes Vision, a single refractive surgery center in Draper, UT, USA. All surgeries were conducted by a single surgeon (PH) between February 2022 and September 2025. The study adhered to the tenets of the Declaration of Helsinki and received Institutional Review Board approval by the Biomedical Research Alliance of New York (#A20-12-547-823). Informed consent was obtained for the use of deidentified patient data.

Eyes were eligible if (1) the patient underwent primary myopic LASIK and (2) completed preoperative visual acuity, refraction, and Pentacam examination (Oculus Cornea OCT, Oculus Optikgeräte GmbH, Wetzlar, Germany), and (3) completed at least one postoperative refraction and visual acuity between 60 and 120 days. Exclusion criteria included prior corneal refractive surgery, corneal disease, or ocular comorbidities that limit refractive accuracy (e.g., glaucoma, age-related macular degeneration, or keratoconus). 

All procedures were carried out using the Zeiss VisuMax 500 femtosecond laser (Carl Zeiss Meditec AG, Jena, Germany) and the WaveLight EX500 excimer laser (Alcon Laboratories Inc., Fort Worth, Texas, USA). Flaps were created with a diameter of 8.2 mm and an intended thickness of 110 µm. All treatments used the wavefront-optimized profile, with the optical zone set to 6.5 mm in all eyes and a transition zone extending the total ablation diameter to 9.0 mm.

Throughout this paper, "pupil center" refers to the center of the entrance pupil, which is the virtual image of the anatomical pupil formed by the cornea. The corneal apex, the point of maximum anterior corneal curvature, was not used in this study. "Corneal vertex" refers to the point on the anterior corneal surface intersected by the observation axis when the subject fixates coaxially with that axis and is used interchangeably with the subject-fixated coaxially sighted corneal light reflex, which marks that point clinically. In the current study, chord mu refers to the distance between the pupil center and the corneal vertex [11].

The groups were determined by the centration setting entered on the laser. Eyes treated with no centration offset (centered on the entrance pupil) were assigned to the pupil-centered group, and eyes treated with any non-zero offset toward the corneal vertex were assigned to the offset group. The laser's eye tracker follows the entrance pupil rather than the corneal light reflex, so the offset was delivered as a displacement applied relative to the tracked pupil center. For offset eyes, the laser measured the pupil-to-vertex offset intraoperatively and was set to displace the ablation by 50% of that measurement [3]. The laser platform, flap parameters, nomogram, and surgical technique were unchanged across the study period.

Preoperative tomography was performed with Pentacam imaging. With the patient fixating the internal target, the Scheimpflug camera aligns its axis of rotation to the corneal vertex and reports the displacement of the entrance pupil center from that axis in Cartesian coordinates. Chord mu was taken as the magnitude of this vector and is distinct from the intraoperative measurement used by the laser to set the treatment offset. Horizontal coordinates were converted to right-eye format by negating the x value for left eyes so that nasal displacement carries the same sign in both eyes. Keratometry and central corneal thickness were taken from the same examination. 

Refractive accuracy was assessed as the miss from target, defined as postoperative minus target spherical equivalent, both signed and in absolute value, together with the proportions within ±0.50 D and ±1.00 D of target. Astigmatic outcome was assessed as the magnitude of residual refractive cylinder. Astigmatism was also assessed as a vector using Thibos power vectors J0 and J45. Alpine vector analysis was performed with the preoperative, target, and achieved astigmatism, yielding the target-induced astigmatism vector (TIA), the surgically induced astigmatism vector (SIA), and the difference vector (DV) [12]. These vector calculations were implemented in Python (Python Software Foundation, Wilmington, DE) according to the formulas published by Alpins [12]; no proprietary vector-analysis software was used. Efficacy and safety indices were calculated. Eyes with a planned monovision target, defined as any non-zero target sphere, were excluded from the efficacy index and uncorrected acuity endpoints. 90 eyes were excluded on this basis.

Statistical analyses were performed using Microsoft Excel (v16.106; Microsoft Corporation, Redmond WA, USA) and Python 3.11.7 using statsmodels 0.14.6, pandas 3.0.0, NumPy 2.4.1, and Matplotlib 3.10.8. Because most patients contributed both eyes, all comparisons used generalized estimating equations (GEE) clustered on the patient, with an independent working correlation and robust standard errors. Binary outcomes, such as percent within ±0.50 D of target refraction, were intentionally modeled with a linear probability model so that the coefficient is a risk difference in percentage points rather than an odds ratio. The astigmatism centroids were compared with a joint two-degree-of-freedom Wald test on the J0 and J45 components, estimated in a single GEE with both vector components stacked per eye. Adjusted models included the baseline characteristics that differed between groups at P < 0.05, namely age at surgery, sex, and corneal astigmatism. This selection rule was specified before the outcome analyses were run. To test whether the effect of centration depended on the pupil-to-vertex chord, the primary models were refit with an added term allowing the difference between groups to vary with chord mu. All analyses were complete-case. Eyes lacking a given measurement were excluded from that endpoint alone, and no imputation was performed. Because the analysis was retrospective, no endpoint was designated as primary in advance. All outcomes were treated equally, and no adjustment for multiplicity was applied. Unless stated otherwise, reported differences and confidence intervals (CIs) are from the adjusted models, and a two-sided P < 0.05 was considered statistically significant.

## Results

A total of 389 eyes of 211 patients underwent myopic LASIK between February 2022 and September 2025 and met inclusion criteria. Both eyes were treated in 178 patients and a single eye in 33. 192 eyes of 110 patients were treated with the ablation centered on the entrance pupil and 197 eyes of 105 patients with a 50% offset toward the corneal vertex. Four patients contributed one eye to each group. Mean preoperative spherical equivalent was −3.36 ± 2.04 D (-8.50 D to 0.00 D) in the pupil-centered group and −3.09 ± 1.78 D (-8.12 D to 0.00 D) in the offset group, and the final refraction was obtained a median of 92 days after surgery (Table 1).

**Table 1:** Preoperative demographic and baseline ocular characteristics Values are mean ± SD or n (%). Comparisons by generalized estimating equations with an independence working correlation and cluster-robust standard errors, clustered on patient. 389 eyes of 211 patients; 178 patients contributed both eyes. Pupil-centered: 192 eyes/110 patients, offset: 197 eyes/105 patients. Age was computed from date of birth and procedure date. *Statistically significant (P < 0.05); CDVA: corrected distance visual acuity

Characteristic	Pupil-centered (centration = 0) n = 192 eyes	Offset (centration ≠ 0) n = 197 eyes	P-value
Patient characteristics			
Age at surgery, years	44 ± 6 (33 to 57)	42 ± 5 (35 to 55)	<0.01*
Male sex	109 (56.8%)	81 (41.1%)	0.02*
Right eye (OD)	100 (52.1%)	103 (52.3%)	0.91
Preoperative refraction			
Sphere, D	-2.86 ± 2.08 (-8.00 to 1.25)	-2.55 ± 1.84 (-7.50 to 2.50)	0.24
Cylinder, D	-0.99 ± 0.78 (-3.50 to 0.00)	-1.09 ± 1.01 (-6.50 to 0.00)	0.39
Spherical equivalent, D	-3.36 ± 2.04 (-8.50 to 0.00)	-3.09 ± 1.78 (-8.12 to 0.00)	0.31
CDVA, logMAR	0.00 ± 0.03 (-0.12 to 0.10)	0.00 ± 0.04 (-0.12 to 0.22)	0.84
Ocular biometry			
Scotopic pupil diameter, mm	5.62 ± 0.99 (3.40 to 8.30)	5.66 ± 0.95 (3.00 to 8.40)	0.72
Central corneal thickness, µm	553.10 ± 29.03 (462.00 to 635.00)	550.03 ± 30.10 (444.00 to 647.00)	0.45
K1 (flat), D	43.46 ± 1.64 (38.40 to 46.80)	43.28 ± 1.39 (38.80 to 46.40)	0.4
K2 (steep), D	44.51 ± 1.51 (39.80 to 48.00)	44.58 ± 1.43 (39.10 to 48.60)	0.73
Mean keratometry, D	43.99 ± 1.54 (39.10 to 47.15)	43.93 ± 1.33 (38.95 to 46.70)	0.79
Corneal astigmatism (K2-K1), D	1.05 ± 0.70 (0.10 to 3.60)	1.30 ± 0.94 (0.00 to 5.90)	0.02*
Chord mu, µm	208.89 ± 111.76 (14.14 to 690.51)	211.21 ± 105.86 (10.00 to 626.50)	0.87
Planned monovision target	49 (25.5%)	41 (20.8%)	0.19

Baseline characteristics are shown in Table 1, and three characteristics differed between groups. Patients in the offset group were significantly younger (42 ± 5 versus 44 ± 6 years; P < 0.01), a smaller proportion were male (81 eyes, 41.1% versus 109 eyes, 56.8%; P = 0.02), and they had greater corneal astigmatism (1.30 ± 0.94 versus 1.05 ± 0.70; D; P = 0.02). These three variables were carried into the adjusted models for outcome analyses.

The groups were otherwise well matched in terms of preoperative sphere, cylinder, spherical equivalent, corrected distance visual acuity, scotopic pupil diameter, central corneal thickness, keratometry, laterality, and the proportion of eyes with a planned monovision target. Notably, chord mu was almost identical between groups (208.89 ± 111.76 µm for the pupil-centered versus 211.21 ± 105.86 µm for the offset group; P = 0.87), with an average of 210.1 ± 108.7 µm (Table 1). Therefore, eyes in the offset group were not chosen on the basis of having a larger chord mu. The displacement actually delivered in the offset group, as recorded by the laser, averaged 116.4 ± 58.4 µm (median 109.1 µm; range 10 to 333 µm).

Postoperative outcomes are shown in Table 2. With the exception of a marginal difference in the Alpins magnitude of error, no endpoint differed significantly between groups. The efficacy index for the pupil-centered and offset group was 1.00 ± 0.15 versus 0.99 ± 0.16 (P = 0.72), respectively, and postoperative uncorrected distance visual acuity in logMAR was 0.00 ± 0.06 for pupil-centered versus 0.00 ± 0.07 for offset (P = 0.68) (Figure 1). The safety index was 1.05 ± 0.13 versus 1.05 ± 0.13 (P = 0.95), and postoperative corrected distance visual acuity was −0.02 ± 0.05 versus −0.02 ± 0.05 logMAR (P = 0.90) (Figure 2). No eye in either group lost two or more lines of corrected distance visual acuity. Refractive accuracy was also high (Figure 3).

**Table 2:** Postoperative refractive, visual and astigmatic outcomes Values are mean ± SD or %. All comparisons were by generalized estimating equations with an independence working correlation and cluster-robust standard errors, clustered on patient. Differences, confidence intervals and P values are adjusted for the baseline characteristics that differed between groups: age, sex and corneal astigmatism. Efficacy index and postoperative UDVA exclude planned-monovision eyes (n = 90; 143 pupil-centered and 156 offset eyes contribute). Difference is offset minus pupil-centered; for percentages, it is a risk difference in percentage points. The vector row compares the two astigmatism centroids in Thibos power-vector space by a joint 2-degree-of-freedom Wald test. Differences are the centroid separation in diopters of cylinder magnitude. *Statistically significant (P < 0.05); UDVA: uncorrected distance visual acuity; CDVA: corrected distance visual acuity

Outcome	Pupil-centered (centration = 0) n = 192 eyes	Offset (centration ≠ 0) n = 197 eyes	Difference	95% CI	P-value
Astigmatism					
Residual refractive astigmatism, D	0.30 ± 0.29	0.30 ± 0.32	-0.04	-0.10, 0.03	0.27
Astigmatism centroid (J0, J45)	0.20 D @ 177°	0.18 D @ 175°	0.05	—	0.32
Refractive accuracy					
Miss from target, signed, D	-0.05 ± 0.29	-0.08 ± 0.30	0.00	-0.07, 0.06	0.95
Miss from target, absolute, D	0.22 ± 0.19	0.22 ± 0.21	0	-0.05, 0.04	0.84
Within ±0.50 D of target, %	94.8% (182)	92.9% (183)	-1.44	-6.28, 3.41	0.56
Within ±1.00 D of target, %	100.0% (192)	100.0% (197)	—	—	—
Efficacy					
Efficacy index	1.00 ± 0.15	0.99 ± 0.16	-0.01	-0.05, 0.03	0.72
Postoperative UDVA, logMAR	0.00 ± 0.06	0.00 ± 0.07	0	-0.01, 0.02	0.68
Safety					
Safety index	1.05 ± 0.13	1.05 ± 0.13	0	-0.03, 0.04	0.95
Postoperative CDVA, logMAR	-0.02 ± 0.05	-0.02 ± 0.05	0.00	-0.01, 0.01	0.9

**Figure 1:**
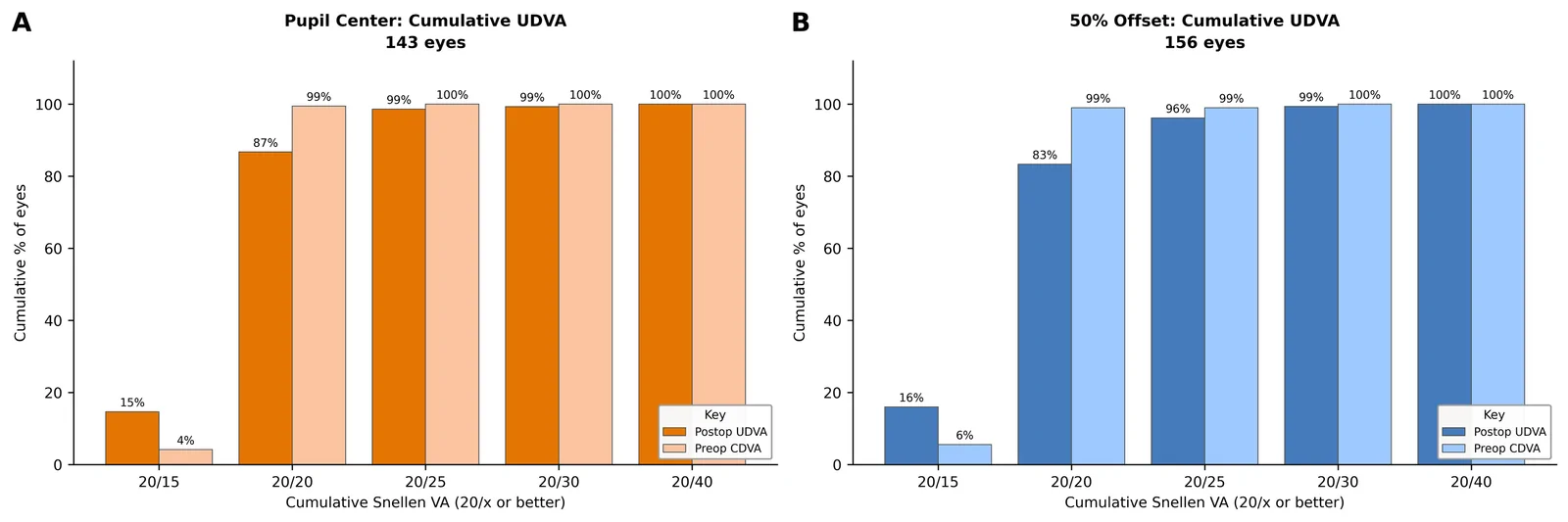
Cumulative Snellen uncorrected distance VA at three months Cumulative proportion of eyes achieving each Snellen acuity level or better, comparing postoperative UDVA against preoperative CDVA. (A) Pupil-centered eyes (n = 143; 49 eyes with a planned monovision target excluded). (B) 50% offset eyes (n = 156; 41 monovision eyes excluded). UDVA: uncorrected distance visual acuity; CDVA: corrected distance visual acuity; VA: visual acuity.

**Figure 2:**
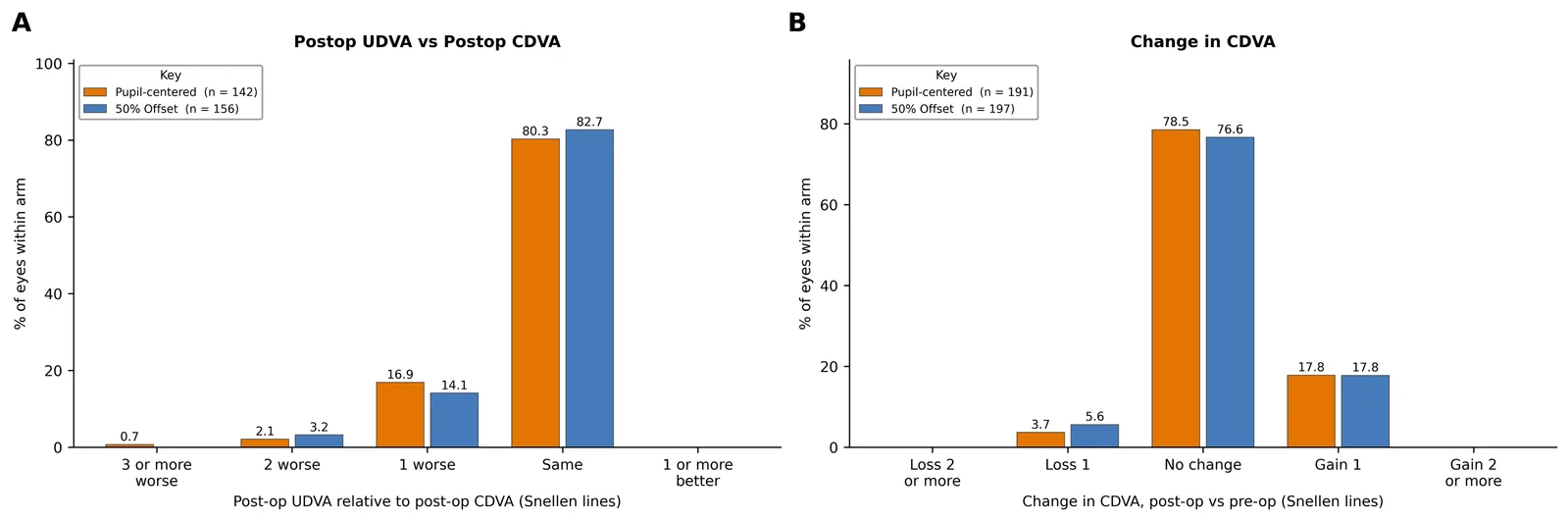
Postoperative visual acuity relative to corrected acuity and change in corrected acuity (A) Postoperative UDVA relative to postoperative CDVA in Snellen lines (pupil-centered n = 142, 50% offset n = 156; 90 eyes with a planned monovision target excluded). (B) Change in CDVA from preoperative to postoperative in Snellen lines, all eyes with paired measurements (pupil-centered n = 191, 50% offset n = 197). UDVA: uncorrected distance visual acuity; CDVA: corrected distance visual acuity.

**Figure 3:**
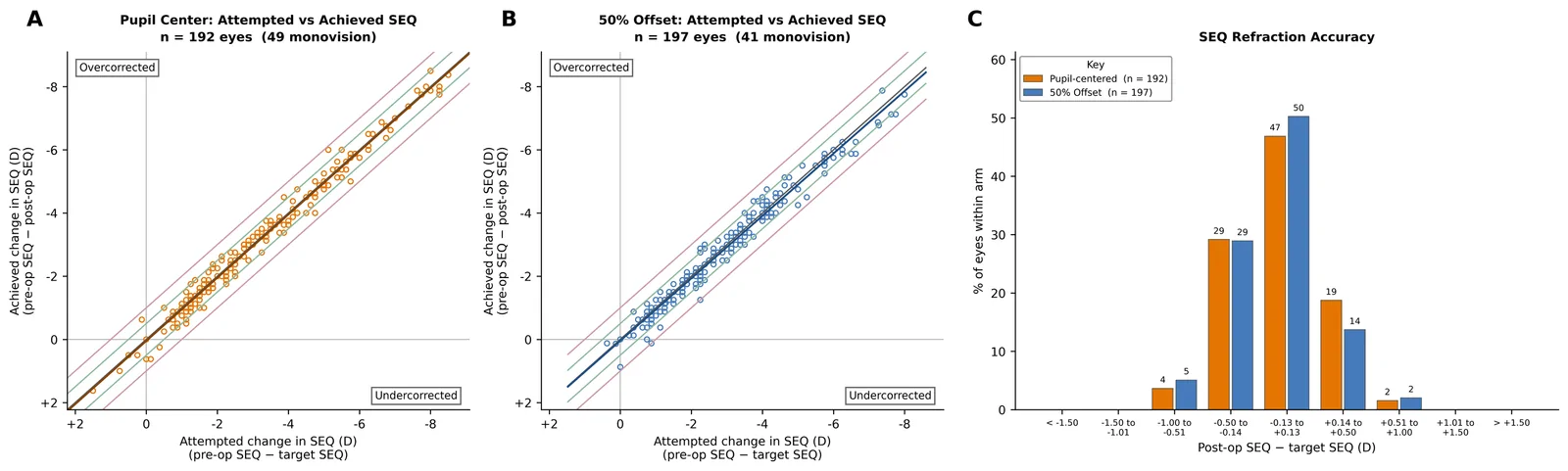
Attempted versus achieved spherical equivalent correction and refractive accuracy (A, B) Attempted versus achieved change in SEQ for pupil-centered eyes (A, n = 192, including 49 monovision eyes) and 50% offset eyes (B, n = 197, including 41 monovision eyes). The central diagonal marks perfect agreement, flanking lines mark ±0.50 D and ±1.00 D, and the colored line is the least-squares fit; points above the diagonal are overcorrected and points below are undercorrected. Both panels share one scale. (C) Distribution of postoperative SEQ minus target SEQ, as the percentage of eyes within each treatment arm. SEQ: spherical equivalent; D: diopters; n: number of eyes.

Residual refractive astigmatism was 0.30 ± 0.29 D in the pupil-centered group and 0.30 ± 0.32 D in the offset group (difference −0.04 D, 95% CI −0.10 to 0.03; P = 0.27) (Table 2, Figure 4). Analyzed as a vector, the astigmatism centroids were 0.20 D at 177° and 0.18 D at 175° for pupil-centered and offset groups, respectively, and did not differ (P = 0.32) (Table 2, Figure 5).

**Figure 4:**
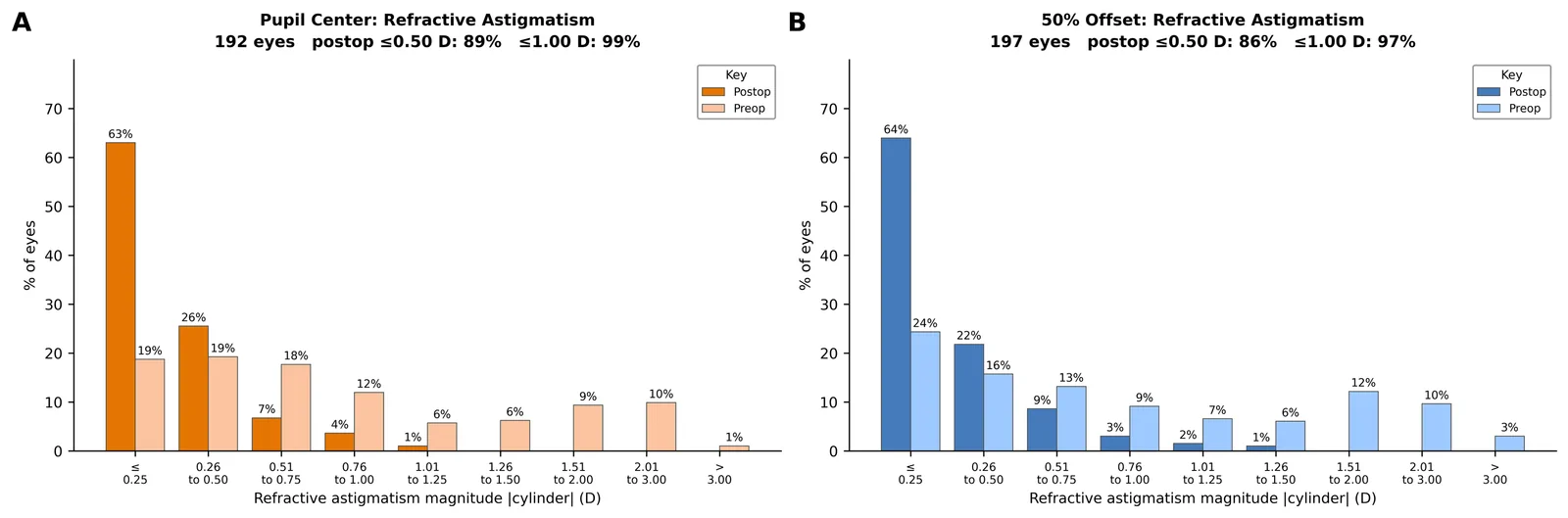
Refractive astigmatism magnitude before and after surgery Distribution of refractive astigmatism magnitude (absolute cylinder) before and after surgery for (A) pupil-centered eyes (n = 192) and (B) 50% offset eyes (n = 197). Postoperatively, 89% of pupil-centered and 86% of offset eyes were 0.50 D or less, and 99% and 97% were 1.00 D or less, respectively. Both panels share one scale. D: diopters; n: number of eyes.

**Figure 5:**
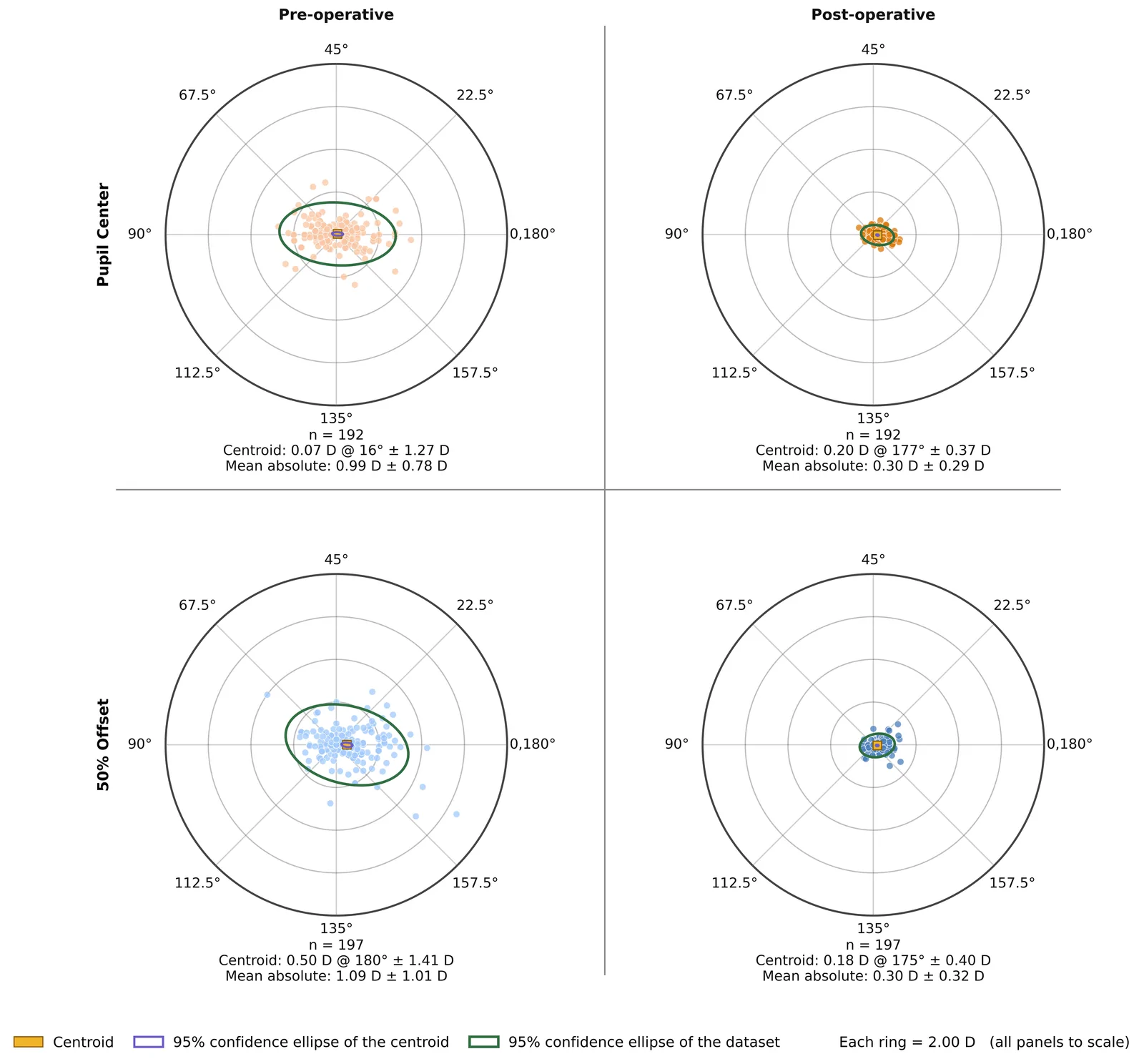
Double-angle plots of preoperative and postoperative manifest refractive astigmatism Double-angle plots for pupil-centered eyes (top row; n = 192) and 50% offset eyes (bottom row; n = 197), before surgery (left column) and after surgery (right column). Each point is one eye; the gold marker is the group centroid, the inner ellipse is the 95% confidence ellipse of the centroid, and the outer ellipse is the 95% confidence ellipse of the dataset. Each ring represents 2.00 D, and all four panels are drawn to one scale. The caption beneath each panel reports the number of eyes, the centroid with its standard deviation, and the mean absolute astigmatism with its standard deviation. D: diopters; n: number of eyes.

The Alpins magnitude of error was significantly different between groups: −0.05 ± 0.32 versus −0.04 ± 0.32 D (difference 0.065 D, 95% CI 0.002 to 0.127; P = 0.043) for pupil-center and offset, respectively. The 46 eyes with no preoperative cylinder were excluded from ratio-based indices, leaving 170 pupil-centered and 173 offset eyes. The correction index was 0.96 ± 0.37 in the pupil-centered group versus 0.99 ± 0.39 in the offset group (difference 0.07, 95% CI −0.01 to 0.16; P = 0.10), and the absolute angle of error was 8.1 ± 13.0° versus 10.0 ± 19.4° (P = 0.24) for pupil-centered and offset groups, respectively. Because the target cylinder was zero in all eyes, the target-induced astigmatism vector is equal to the preoperative astigmatism, and the difference vector is equal to the residual astigmatism, both of which are reported above. The surgically induced astigmatism was 0.95 ± 0.73 D in the pupil-centered group and 1.06 ± 0.97 D in the offset group. The remaining Alpins indices did not differ between groups, with an index of success of 0.37 ± 0.39 versus 0.41 ± 0.63 (P = 0.42), a flattening index of 0.88 ± 0.44 versus 0.79 ± 0.60 (P = 0.28), an absolute torque of 0.15 ± 0.16 versus 0.15 ± 0.20 D (P = 0.46), and a signed angle of error of 3.4 ± 14.9 versus 3.1 ± 21.6 degrees (P = 0.90) for pupil-centered and offset groups, respectively.

Refractive accuracy was similarly comparable. The signed miss from target was −0.05 ± 0.29 D versus −0.08 ± 0.30 D (difference 0.00 D, 95% CI −0.07 to 0.06; P = 0.95) and the absolute miss 0.22 ± 0.19 D versus 0.22 ± 0.21 D (difference 0.00 D, 95% CI −0.05 to 0.04; P = 0.84), for pupil-centered and offset groups, respectively. Within ±0.50 D of target were 182/192 (94.8%) of the eyes for the pupil-centered group and 183/197 (92.9%) of the eyes for the offset group (difference −1.44 percentage points, 95% CI −6.28 to 3.41; P = 0.56) (Table 2, Figure 3). All eyes in both groups were within ±1.00 D.

Because centration might be expected to have a greater impact on eyes with a larger pupil-to-vertex chord, the present study examined if differences between groups changed with chord mu. No interactions approached significance (residual astigmatism P = 0.51; absolute miss from target P = 0.49; efficacy index P = 0.77; safety index P = 0.46), providing no evidence that the effect of centration depended on the magnitude of chord mu.

## Discussion

In this retrospective cohort of 389 myopic eyes from 211 patients, no statistically significant difference was detected between ablation centered midway between the pupil center and the corneal vertex and pupil-centered treatment in any refractive or visual outcome, with the exception of a marginal difference in the Alpins magnitude of error (adjusted difference 0.065 D, 95% CI 0.002 to 0.127; P = 0.043). Given our study’s 17 comparisons without multiplicity correction and a confidence interval reaching 0.002 D, this isolated finding should be interpreted with caution. Residual refractive astigmatism, accuracy relative to target refraction, efficacy indices, and safety indices showed no statistically significant differences between groups. Although the confidence intervals for these endpoints were narrow, the study was not designed as an equivalence or non-inferiority trial and no equivalence margins were pre-specified; these findings therefore indicate an absence of detected differences rather than demonstrated equivalence.

Our results align with pooled evidence on visual acuity, but diverge on refractive outcomes. Xu et al’s meta-analysis of 13 studies found no difference between vertex and pupil-referenced centration in the proportion achieving 20/20 uncorrected vision, in loss of two or more lines of corrected acuity, or in postoperative astigmatism [10]. In the present cohort, 124/143 (86.7%) of pupil-centered and 130/156 (83.3%) of offset eyes achieved 20/20 or better uncorrected acuity, no eye in either group lost two or more lines of corrected acuity, and residual astigmatism was identical (Table 2, Figure 1, Figure 2B, Figure 4). The proportion of eyes within ±0.50 D of the target refraction favored the vertex-centered group in Xu's pooled analysis; however, we found no such advantage: 182/192 (94.8%) versus 183/197 (92.9%) (P = 0.56) for pupil-centered vs offset, respectively (Table 2, Figure 3C) [10]. Xu et al. reported this advantage as an odds ratio of 1.49. Applied to the 94.8% accuracy of our pupil-centered group, this corresponds to a benefit of approximately 1.7 percentage points, which is well within our confidence interval and therefore not excluded by our data (Table 2) [10]. Although significant endpoints differ between the present study and Xu et al’s pooled analysis, comparisons must be made with caution due to the heterogeneity of each included study’s design. The paper’s “vertex-centered” groups ranged from 50% offset towards the vertex to 100%, and follow-up durations ranged from 1 to 6 months [10]. 

The study closest in design to the current study, and the only other comparison of pupil centration against a 50% offset, is that of Kermani et al [8]. Their findings differ from our study as the offset group achieved better refractive predictability (81% versus 64% within ±0.50 D of target), alongside a nominally higher proportion losing two or more lines of corrected acuity (10% versus 6%) (Table 2, Figure 2B, Figure 3C) [8]. However, three factors could contribute to this difference. Their cohort was hyperopic, with a mean pupil-to-vertex chord of approximately 0.50 mm, which is more than twice the current study’s [8]. Thus, Kermani et al’s 50% offset displaced the ablation by roughly 0.25 mm against approximately 0.11 mm in our cohort [8]. Their groups were also markedly unequal (118 pupil-centered versus 52 offset analyzed at three months), and no statistical comparisons were reported [8].

The study by Okamoto et al. is similar in design, though it centered the ablation at 80% of the pupil-to-reflex distance [2,9]. Their cohort resembles ours closely, with a mean pupil-to-reflex distance of 0.20 mm against our mean chord mu of 0.21 mm (Table 1) [2]. However, their significant findings of improved efficacy and safety differed from our null results. The most obvious explanation for this discrepancy is that a 50% offset is simply too small to see the benefit Okamoto et al. reported. An 80% offset leaves the ablation approximately 40 µm from the vertex, whereas the 50% offset used in the present study left it approximately 116 µm away. If displacement toward the vertex drove the outcome, full centration should outperform 80%; however, current literature does not support this. A randomized trial of 420 myopic eyes [13] and a cohort of 258 eyes [14], both centered entirely on the vertex, found no difference from pupil-centered treatment in uncorrected or corrected acuity, in spherical equivalent, or in the efficacy and safety indices. The magnitude-dependent effect also fails within our own data, where the effect of centration did not vary with chord mu on any endpoint. 

The conflicting published outcomes are more likely attributable to differences in study design rather than to the magnitude of the offset. Indeed, Okamoto et al. reported using an optical zone setting of 4.5 mm compared to the present study’s 6.5 mm [2]. A 4.5 mm zone falls short of modern recommendations that the optical zone should equal or exceed the functional entrance pupil to avoid glare and haloes [7,15]. When the zone is smaller than the dilated pupil, uncorrected cornea overlaps part of the pupil even with perfect centration. Any displacement of the zone moves that uncorrected region across the visual axis, causing outcomes to be acutely sensitive to the choice of centration point [2,7,16]. By contrast, a 6.5 mm zone leaves nearly half a millimeter of corrected margin around a typical scotopic pupil, resulting in little room for centration differences to be detected, which may explain our null results. Alternatively, the eye's own tolerance for optical imperfections may account for the absence of an effect. Even when zone coverage is imperfect, the displacements compared in the present study still fall below the levels at which visual harm has been demonstrated. In an early topographic analysis of PRK, uncorrected acuity was preserved for ablation decentrations of up to 1.00 mm, and in a more recent series of SMILE patients, 99% of eyes achieved 20/20 or better uncorrected acuity despite a mean treatment decentration of 0.17 mm, with all eyes within 0.50 mm [17,18]. The approximately 116 µm of separation between our two centration methods is smaller still, suggesting that our groups sat comfortably within the eye's optical tolerance.

This study has several limitations. It is retrospective and non-randomized, which may limit generalizability. Centration strategy changed at a single point in the surgeon's practice; thus, group membership is effectively determined by the period of treatment. Because period is collinear with centration, it cannot be adjusted for, and the resulting confounding is not one that statistical adjustment can remove. The surgeon, laser platforms, flap parameters, zone settings, nomogram, surgical technique, and pre-operative measurement protocol were unchanged across the study period, and eligibility criteria were applied uniformly to both periods. We cannot confirm, however, that patient selection, referral patterns, perioperative management, laser software, or the surgeon's experience remained constant over three and a half years. These results should therefore be read as the absence of a detectable difference between two periods of practice that differed principally in centration, rather than as isolating centration itself. The three differences at baseline, age, sex, and corneal astigmatism, were adjusted for in all outcome models; however, the adjustment cannot rule out unmeasured confounding variables. Postoperative corneal and ocular aberrations were not measured, so the endpoints where vertex centration most consistently shows benefit fall outside this study. No patient-reported outcomes such as glare or night vision disturbance were collected. Follow-up was a median of 92 days at a single postoperative visit, so refractive stability could not be assessed. Finally, while the cohort was adequately sized for its primary comparisons, it was underpowered for subgroup analysis. Whether centration affects outcomes in eyes with a large chord mu or in eyes whose scotopic pupil approaches the optical zone could not be reliably assessed and remains an open question.

## Conclusions

In this cohort of 389 myopic LASIK eyes, no statistically significant differences in refractive accuracy, residual astigmatism, or corrected and uncorrected visual acuity were detected between pupil-centered ablation and ablation centered midway between the entrance pupil and the corneal vertex. These findings are limited to standard high-contrast measures of refractive and visual outcome at three months, at the chord mu values typically seen in myopic eyes. Contrast sensitivity, glare, night vision disturbance, and post-operative aberrations were not assessed. The study was not designed as an equivalence trial, and no equivalence margins were pre-specified, so the absence of detected differences does not establish that the two strategies are equivalent. Whether a difference exists for larger offsets, for eyes with a large chord mu, for aberrometric endpoints, or for patient-reported quality of vision remains to be established by prospective randomized comparison.
